# Proton pump inhibitors and chronic kidney disease risk: a comparative study with histamine-2 receptor antagonists

**DOI:** 10.1038/s41598-023-48430-9

**Published:** 2023-12-01

**Authors:** Takhyeon Kweon, Yerim Kim, Kyung Joo Lee, Won-Woo Seo, Seung In Seo, Woon Geon Shin, Dong Ho Shin

**Affiliations:** Department of Internal Medicine, College of Medicine, Kangdong Sacred Heart Hospital, Hallym University, 150, Seongan-to, Guangdong-Gu, Seoul, 05355 Korea

**Keywords:** Diseases, Nephrology

## Abstract

This observational study explored the association between proton pump inhibitor (PPI) and histamine-2 receptor antagonist (H2RA) use and the risk of chronic kidney disease (CKD). Using the National Health Insurance Service-National Sample Cohort (NHIS-NSC) and six-hospital electronic health record (EHR) databases, CKD incidence was analyzed among PPI and H2RA users. Propensity score matching was used to balance baseline characteristics, with 1,869 subjects each in the PPI and H2RA groups from the NHIS-NSC, and 5,967 in EHR databases. CKD incidence was similar for both groups (5.72/1000 person-years vs. 7.57/1000 person-years; HR = 0.68; 95% CI, 0.35–1.30). A meta-analysis of the EHR databases showed no significant increased CKD risk associated with PPI use (HR = 1.03, 95% CI: 0.87–1.23). These results suggest PPI use may not increase CKD risk compared to H2RA use, but the potential role of PPI-induced CKD needs further research. Clinicians should consider this when prescribing long-term PPI therapy.

## Introduction

Proton-pump inhibitors (PPIs) are one of the most commonly prescribed medications worldwide, with millions of people using them to manage gastroesophageal reflux disease and peptic ulcers. PPIs irreversibly inhibit the H + /K + -ATPase enzyme in the stomach, thereby decreasing acid secretion^1^. While PPIs are effective in treating these conditions, recent studies have raised concerns about potential adverse effects associated with their long-term use. These adverse effects include hypomagnesemia^2,3^, acute kidney injury (AKI)^4,5^, and acute tubular interstitial nephritis (ATIN)^6–8^. There is also evidence suggesting a correlation between PPI use and chronic kidney disease (CKD)^9–11^, although the precise mechanism is not well understood.

CKD is a global public health problem affecting millions of people worldwide, and it is associated with significant morbidity, mortality, and healthcare costs^12–15^. Previous epidemiologic studies have shown a potential link between PPI use and an increased risk of developing CKD. However, these studies have several limitations, including higher rates of comorbidities in the PPI group compared to the control group, lack of important baseline CKD-related information such as estimated glomerular filtration rate (eGFR) and concomitant medication, limited data on the duration of PPI use, and multiple medication switches during the observation period ^9–11^. Addressing these limitations is crucial to better understand the potential risks associated with long-term PPI use and inform clinical decision-making.

To address these limitations of observational studies, a distributed research network using a Common Data Model (CDM) has been developed to standardize heterogeneous data sources into a consistent format using the CDM-based vocabulary provided by the Observational Health Data Sciences and Informatics (OHDSI) organization^16,17^. This enables researchers to conduct clinical research using standardized, large-scale data.

This study seeks to evaluate the relationship between long-term PPI use and the risk of CKD by using National Health Insurance Service-National Sample Cohort (NHIS-NSC) and multicenter electronic health record (EHR) database, which have been converted into the Observational Medical Outcomes Partnership Common Data Model (OMOP-CDM) format using validated large-scale data. Specifically, our study aims to investigate the incidence of CKD in new users of acid suppression therapy, either PPI or histamine H2 receptor antagonist (H_2_RA) and compare the risk of CKD between these two groups. By addressing the limitations of previous studies and using a standardized, large-scale database, our study aims to provide a more robust understanding of the potential link between PPI use and CKD. This, in turn, may inform clinical decision-making and improve patient outcomes.

## Results

### Study flow and baseline characteristics

The NHIS-NSC CDM database included 1,125,700 subjects from 2002 to 2013, while the six-hospital CDM databases encompassed 10,083,608 subjects from 1999 to 2018. To balance the baseline characteristics between the PPI and H_2_RA groups, PSM was performed in these databases. Supplementary Table S1 provides additional information about each database and its specific study periods.

**Figure 1:**
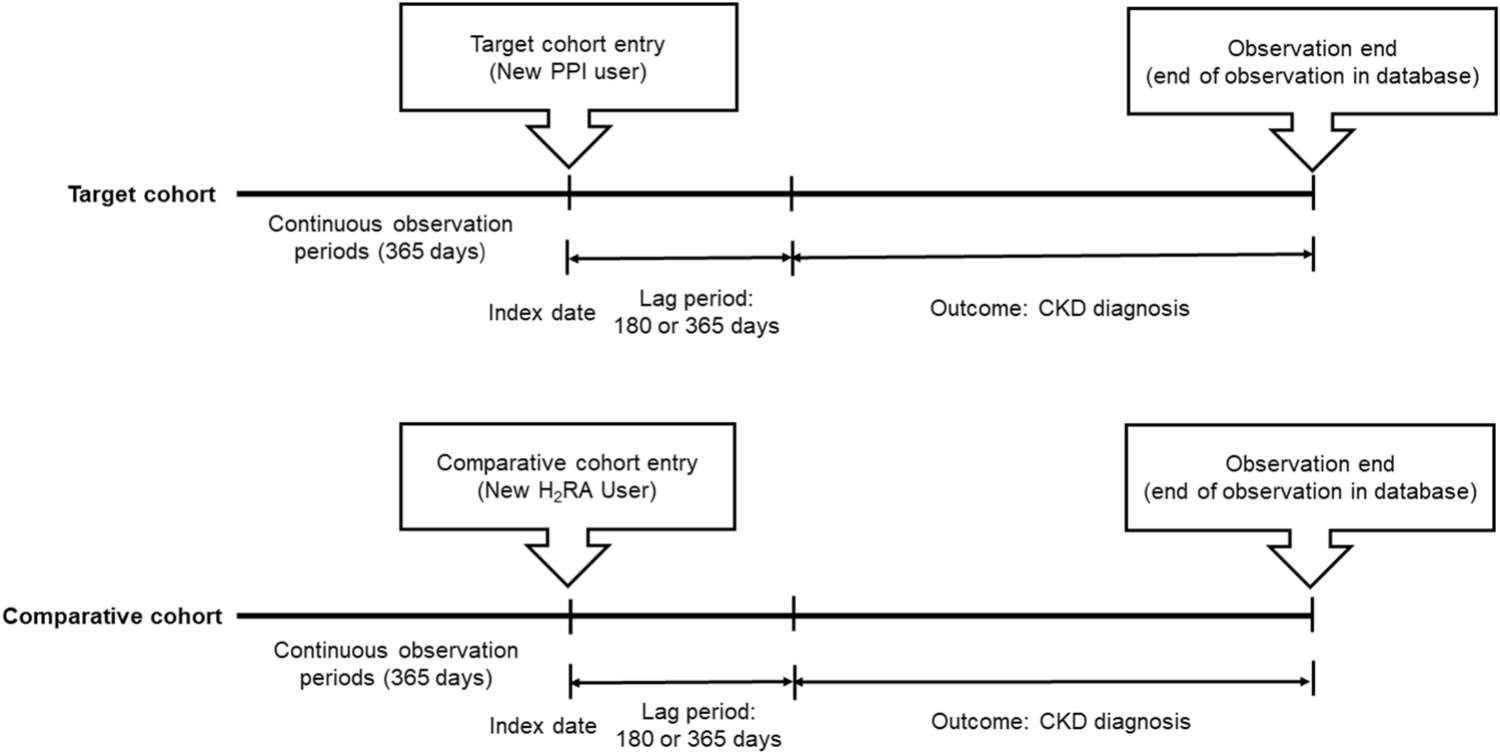
Diagram of cohort construction. PPI, proton pump inhibitor; H_2_RA, histamine 2 receptor antagonist.

**Figure 2:**
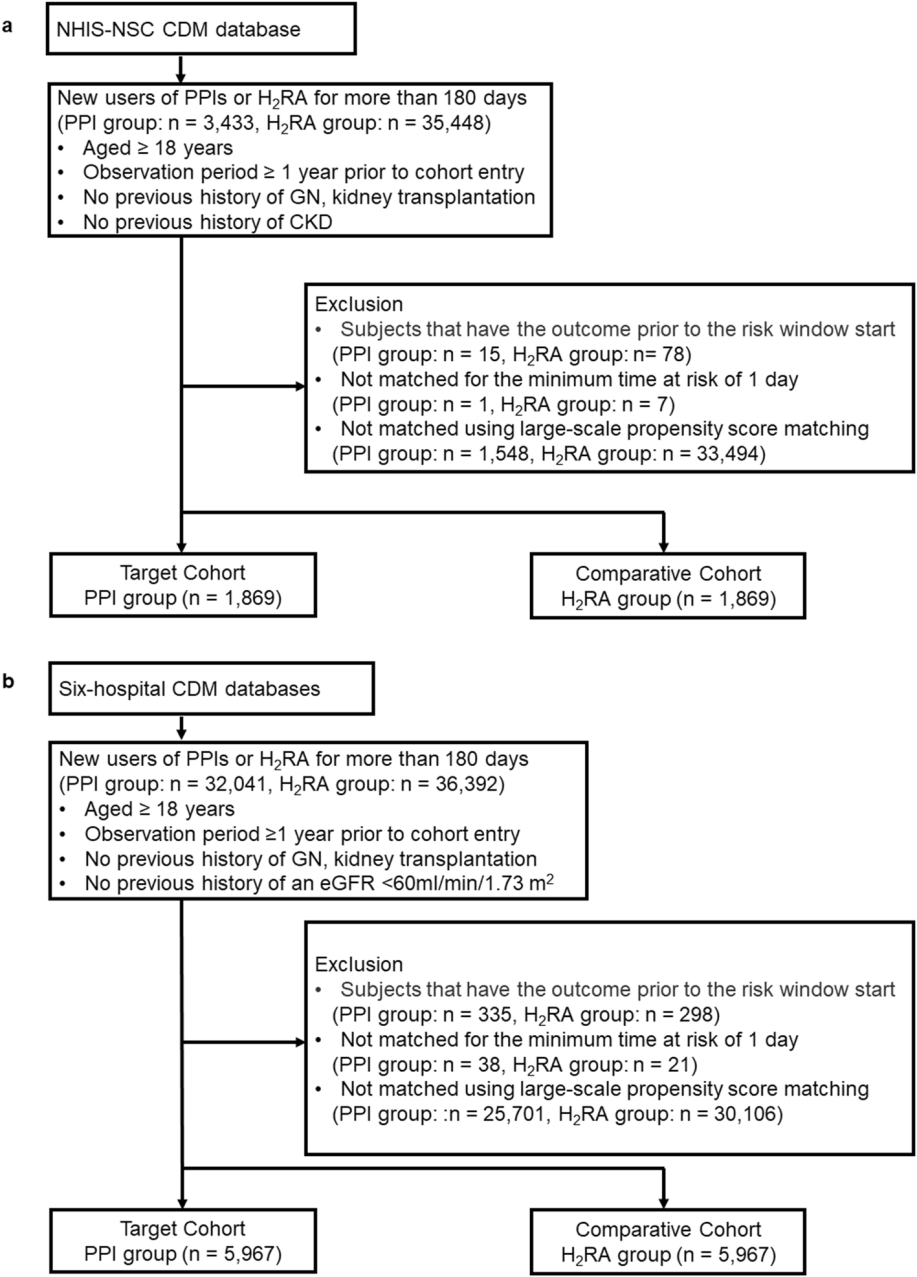
Flowchart of Study Participants in NHIS-NCS CDM database (**a**) and six hospital-based CDM databases (**b**). NHIS-NSC CDM, National Health Insurance Service-National Sample Cohort Common Data model; GN, glomerulonephritis; CKD, chronic kidney disease.

In the NHIS-CDM database, 38,881 subjects were eligible for inclusion. After applying exclusion criteria and performing PSM with 12,949 covariates, 1,869 subjects were analyzed in both groups, respectively. Similarly, in the six-hospital CDM database, 68,433 subjects were initially included. After applying exclusion criteria and performing PSM with covariates ranging from 4,695 to 6,573, the final analysis was performed on 5,967 subjects in both groups, respectively (Fig. 1). Most standardized mean differences were approximately 0.1 after PSM (Fig. 2). Supplementary Table S2 and Figure S1 presented a list of critical covariates considered in PSM and the relative risks for the negative control outcomes to assess systemic errors, respectively. Supplementary Figure S2 showed the distribution of propensity scores for both study groups in each dataset, before and after matching.

Before PSM, the PPI group in the NHIS-NSC CDM database had a higher Charlson comorbidity index score, gastroesophageal reflux disease, hyperlipidemia, and malignant neoplastic disease compared to the H_2_RA group. However, after PSM, baseline covariates were well-balanced in both groups. Table 1 displays the baseline characteristics of the matched cohorts, including covariates with proportions exceeding 5% of the total patients after PSM. Supplementary Table S4 – Table S9 presents the baseline characters for each six hospital cohorts.

**Table 1:** Baseline characteristics of PPI and H_2_RA groups with ≥ 180 days of use in the NHIS-NSC CDM and six-hospital CDM databases before and after propensity score matching.

	NHIS-NSC CDM					
	Before PS adjustment			After PS adjustment		
	PPIs	H_2_RAs	SMD	PPIs	H_2_RAs	SMD
Characteristic (%)	(*n* = 3,433)	(* n* = 35,449)		(* n* = 1,869)	(* n* = 1,869)	
Age group						
20–24	0.4	0.6	−0.04	< 0.3	0.6	−0.07
25–29	0.9	1	−0.01	1.2	1.2	0
30–34	1.6	1.7	−0.01	1.3	1.8	−0.04
35–39	3.1	2.8	0.02	2.9	2.5	0.03
40–44	6.2	4.5	0.07	4.9	4.2	0.03
45–49	8.6	6.8	0.07	8.3	7.7	0.02
50–54	12.5	9.7	0.09	12.7	12.2	0.02
55–59	13.5	10.9	0.08	13.5	12.9	0.02
60–64	14.8	12.9	0.06	13.5	12.7	0.02
65–69	12.5	14.9	−0.07	12.9	138	−0.03
70–74	10.9	14.5	−0.11	12.3	12.9	−0.02
75–79	7.3	10.5	−0.11	7.7	8.7	−0.04
80–84	4.8	5.8	−0.05	5.6	5.1	0.02
85–89	2	2.5	−0.04	2.1	2.3	−0.02
Sex: female	46.1	62.3	−0.33	51.5	51.4	0
Medical history						
Diabetes mellitus	21.1	22	−0.02	21.1	21.3	0
Gastroesophageal reflux disease	33.7	11.6	0.55	31.1	33.6	−0.05
Hyperlipidemia	41.4	27.2	0.3	38.2	41.0	−0.06
Hypertensive disorder	50.7	49.1	0.03	51.0	51.3	−0.01
Visual system disorder	35.0	38.5	−0.07	36.1	36.7	−0.01
Cerebrovascular disease	7.4	7.8	−0.02	8.2	7.9	0.01
Heart disease	26.8	25.8	0.02	26.2	28.3	−0.05
Ischemic heart disease	16.7	14.2	0.07	16.1	17.7	−0.04
Malignant neoplastic disease	9.5	6.2	0.12	8.9	10.0	−0.04
Medication						
Agents acting on the renin-angiotensin system	30.6	23.9	0.15	29.5	30.7	−0.03
Antibacterials for systemic use	60.1	73.1	−0.28	62.4	62.2	0
Antidepressants	14.3	18.8	−0.12	14.5	15.1	−0.02
Antiepileptics	7.6	11.6	−0.13	8.6	7.7	0.03
Anti−inflammatory and antirheumatic products	54.1	74.2	−0.43	57.6	57.3	0.01
Antineoplastic agents	8.4	5.4	0.12	8.1	8.1	0
Antithrombotic agents	56.1	62.1	−0.12	58.6	58.2	0.01
Drugs for acid-related disorders	27.7	38.2	−0.22	31.0	29.5	0.03
Drugs for obstructive airway diseases	35.8	51.3	−0.32	38.1	36.7	0.03
Drugs used in diabetes	17.6	15.2	0.06	17.4	17.3	0
Lipid modifying agents	31.0	18.5	0.29	28.4	28.8	−0.01
Opioids	43.6	57.3	−0.28	45.9	45.4	0.01
Psycholeptics	51.7	66.9	−0.31	52.0	51.4	0.01

	Six-hospital CDM					
	Before PS adjustment			After PS adjustment		
	PPIs	H_2_RAs	SMD	PPIs	H_2_RAs	SMD
Characteristic (%)	(*n* = 32,041)	(* n* = 36,392)		(* n* = 5,967)	(* n* = 5,967)	
Age group						
20–24	0.6	0.9	−0.03	0.6	0.7	−0.01
25–29	0.8	1	−0.03	1.3	1.1	0.02
30–34	1.0	1.5	−0.04	1.2	1.2	0
35–39	2	2.4	−0.03	2.4	2.4	0
40–44	3.4	4	−0.04	3.4	3.5	0
45–49	5.8	6.2	−0.02	6.1	5.8	0.01
50–54	9.5	9.3	0.01	9.1	8.9	0.01
55–59	13.2	12.0	0.04	12.3	12.6	−0.01
60–64	15.3	13.4	0.05	15.3	14.6	0.02
65–69	14.4	14.2	0.01	16.8	14.1	0.08
70–74	13.6	14.6	−0.03	14.2	14.2	0
75–79	11.3	12	−0.02	9.5	11.5	−0.07
80–84	6.3	5.9	0.02	6.1	6.1	0
85–89	2.2	2.0	0.01	2.3	2.6	−0.02
Sex: female	54.8	55.2	−0.01	53.7	55.5	−0.04
Medical history						
Diabetes mellitus	9.8	9.4	0.02	9.8	9.5	0.01
Gastroesophageal reflux disease	20.7	3.5	0.55	7.3	8.8	−0.05
Hyperlipidemia	19.2	18.1	0.03	16.4	17.3	−0.02
Hypertensive disorder	28	30.2	−0.05	26.9	27.4	−0.01
Visual system disorder	8.4	8.5	0	8.2	7.9	0.01
Cerebrovascular disease	8.1	10.6	−0.09	7.1	6.9	0.01
Heart disease	23.9	22.0	0.05	25.3	26.8	−0.03
Ischemic heart disease	15	12.6	0.07	16.1	16.8	−0.02
Malignant neoplastic disease	9.3	9.2	0.01	12.9	8.6	0.14
Medication						
Agents acting on the renin-angiotensin system	27.2	30.5	−0.07	30.4	29.9	0.01
Antibacterials for systemic use	20.8	28.6	−0.18	21.2	19.9	0.03
Antidepressants	17.3	20	−0.07	16.2	16.3	0.00
Antiepileptics	15.7	18.8	−0.08	14.0	13.4	0.02
Anti-inflammatory and antirheumatic products	58.8	62.0	−0.07	56.0	54.6	0.03
Antineoplastic agents	19.9	15.2	0.12	15	14.6	0.01
Antithrombotic agents	43.6	50.8	−0.15	46.9	46.7	0.01
Drugs for acid−related disorders	32.7	59.6	−0.56	36.0	34.2	0.04
Drugs for obstructive airway diseases	19.1	25.2	−0.15	17.2	16.9	0.01
Drugs used in diabetes	12.5	14.5	−0.06	15.6	13.3	0.07
Lipid modifying agents	36.8	34.5	0.05	37.1	38.0	−0.02
Opioids	32.6	32.7	0.00	30.3	29.4	0.02
Psycholeptics	29.8	35.7	−0.12	29.3	27.8	0.03

NHIS-NSC CDM, National Health Insurance Service-National Sample Cohort Common Data Model; PS, propensity score; PPI, proton pump inhibitor; H_2_RA, H2 receptor antagonist; SMD, standardized mean difference.

Supplementary Table S10 shows the Charlson comorbidity index scores in the NHIS-CDM and six-hospital CDM databases. Overall, the study effectively controlled for covariates and ensured that baseline characteristics were well-balanced between the PPI and H_2_RA groups in both databases.

### PPI use and the risk of CKD

**Figure 3:**
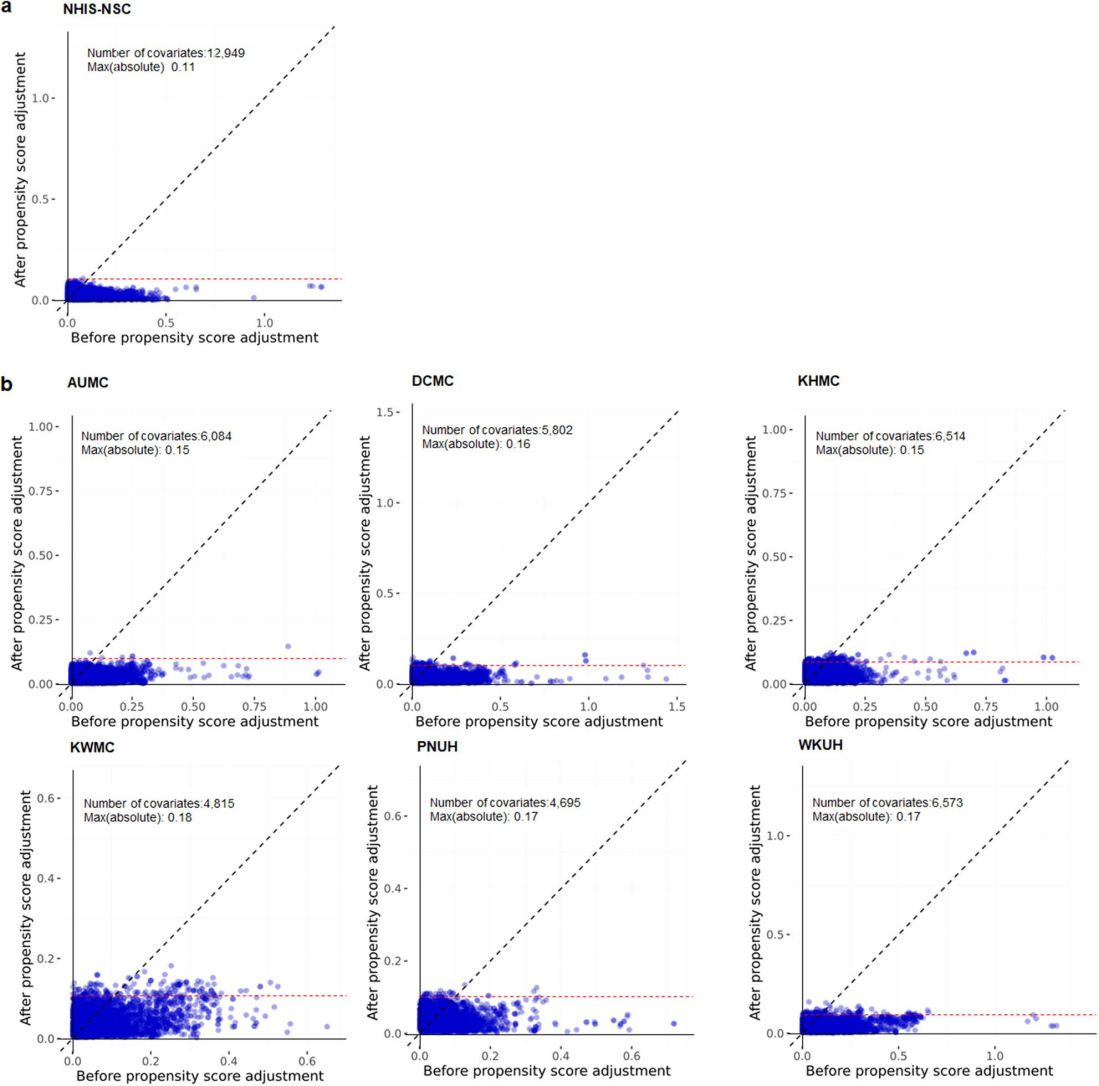
Covariate balance plot before and after propensity score matching in NHIS-NCS CDM database (**a**) and across six hospital-based CDM databases (**b**). NHIS-NSC CDM, National Health Insurance Service-National Sample Cohort Common Data model; AUMC, Ajou University Medical Center; DCMC, Daegu Catholic Medical Center; KHMC, Kyung Hee University Medical Center; KWMC, Kangwon National University Hospital; PUNH, Pusan National University Hospital; WKUH, Wonkwang University Hospital.

The primary analysis in the NHIS-CDM compared the incidence of CKD 180 days after drug exposure between subjects who used PPIs for at least 180 days and those who used H_2_RA for at least 180 days. During a median follow-up of 2.71 years in the PPI group and 2.68 years in the H_2_RA group, 29 patients in the PPI group and 38 patients in the H_2_RA group experienced CKD. The incidence of CKD was comparable between the two groups (5.72/1000 person-years vs. 7.57/1000 person-years, respectively; HR, 0.68; 95% CI, 0.35—1.30, *P* = 0.26). In the secondary analysis, the incidence of CKD was also comparable between the two groups in the six-hospital CDM databases (Table 2). A meta-analysis using the results of the six-hospital CDM databases showed that PPI use was not associated with an increased risk of CKD compared to H_2_RA use (Fig. 3).

**Table 2:** Incidence rates and Cox proportional hazard ratios of chronic kidney disease comparing proton pump inhibitors and histamine-2 receptor antagonists use for ≥ 180 days: NHIS-NSC CDM and six-hospital CDM databases.

Database		Subjects (n)	Person-years	CKD (n)	Incidence rate^a^	HR (95% CI)	P value
NHIS-NSC CDM							
	H_2_RAs ≥ 180 days	1,869	5,018	38	7.57	Ref	
	PPIs ≥ 180 days	1,869	5,066	29	5.72	0.68 (0.35–1.30)	0.26
Six-hospital CDM							
AUMC	H_2_RAs ≥ 180 days	1,472	6,125	82	13.4	Ref	
	PPIs ≥ 180 days	1,472	6,818	94	13.8	1.13 (0.78–1.61)	0.52
DCMC	H_2_RAs ≥ 180 days	766	2,802	64	22.8	Ref	
	PPIs ≥ 180 days	766	2,950	79	26.8	1.26 (0.81–1.96)	0.31
KHMC	H_2_RAs ≥ 180 days	1,125	5,933	124	20.9	Ref	
	PPIs ≥ 180 days	1,125	6,154	138	22.4	1.25 (0.88–1.77)	0.22
KWMC	H_2_RAs ≥ 180 days	723	3,232	136	42.1	Ref	
	PPIs ≥ 180 days	723	3,423	120	35.1	0.82 (0.60–1.13)	0.23
PUNH	H_2_RAs ≥ 180 days	905	2,314	28	12.1	Ref	
	PPIs ≥ 180 days	905	2,457	18	7.3	0.61 (0.31–1.19)	0.15
WKUH	H_2_RAs ≥ 180 days	976	6,305	21	3.3	Ref	
	PPIs ≥ 180 days	976	6,780	34	5.0	1.21 (0.59–2.48)	0.59

NHIS-NSC CDM, National Health Insurance Service-National Sample Cohort Common Data Model; AUMC, Ajou University Medical Center; DCMC, Daegu Catholic Medical Center; KHMC, Kyung Hee University Medical Center; KWMC, Kangwon National University Hospital; PUNH, Pusan National University Hospital; WKUH, Wonkwang University Hospital.

^a^Incidence rate expressed per 1000 person-years.

H_2_RAs, histamine 2 receptor antagonists; PPIs, proton pump inhibitors; HR, hazard ratio; CI, confidence interval; Ref, reference.

### Sensitivity analysis

To further assess the robustness of our results and identify the influence of PPI duration on subjects, we conducted a sensitivity analysis using 1:4 propensity score matching and PPI use ≥ 365 days. The sensitivity analysis results were consistent with the primary analysis, showing no significant difference in the incidence of CKD between the PPI and H_2_RA groups. Additionally, we conducted a subgroup analysis for subjects with PPI use ≥ 365 days, which also found no increased risk of CKD in this subgroup compared to the H_2_RA group (Table 3).

**Table 3:** Sensitivity analysis of proton pump inhibitors and histamine-2 receptor antagonists groups in the NHIS-NSC CDM and six-hospital CDM databases.

PS matching ratio/lag period	NHIS-NSC CDM			Six-hospital CDM		
	HR	95% CI	P-value	HR^a^	95% CI^a^	P-value^a^
≥ 180 days of use						
1:1 matching/180 days	0.68	0.35—1.30	0.26	1.03	0.87—1.23	0.69
1:4 matching/180 days	0.86	0.49—1.46	0.58	1.01	0.88—1.17	0.88
1:1 matching/365 days	0.90	0.47—1.70	0.75	0.97	0.81—1.17	0.75
1:4 matching/365 days	0.91	0.50—1.60	0.76	0.93	0.80—1.09	0.38
≥ 365 days of use						
1:1 matching/180 days	0.71	0.31—1.60	0.42	0.96	0.79—1.17	0.70
1:4 matching/180 days	0.82	0.39—1.61	0.57	1.01	0.85—1.19	0.95
1:1 matching/365 days	1.12	0.43—3.00	0.81	1.19	0.96—1.49	0.11
1:4 matching/365 days	0.80	0.37—1.64	0.56	1.10	0.92—1.32	0.31

NHIS-NSC CDM, National Health Insurance Service-National Sample Cohort-common data model; PS, propensity score.

^a^Results of a six-hospital CDM meta-analysis.

### Subgroup analysis

In the subgroup analysis for patients with diabetic mellitus (DM), both the NHIS-NSC CDM and six-hospital CDM databases showed no significant association between PPI use and CKD risk compared to H_2_RA use across different matching ratios and lag periods (Table 4).

**Table 4:** Subgroup analysis of chronic kidney disease risk comparing proton pump inhibitors and histamine-2 receptor antagonists use for ≥ 180 days in diabetic patients in the NHIS-NSC CDM and six-hospital CDM databases.

PS matching ratio/lag period	NHIS-NSC CDM			Six-hospital CDM		
	HR	95% CI	P-value	HR^a^	95% CI^a^	P-value^a^
1:1 matching/180 days	1.43	0.61—3.51	0.40	1.14	0.72—1.78	0.47
1:4 matching/180 days	1.44	0.71—2.74	0.30	1.13	0.80—1.58	0.79
1:1 matching/365 days	1.51	0.63—3.84	0.38	1.29	0.81—2.06	0.61
1:4 matching/365 days	1.48	0.67—3.15	0.33	1.38	0.92—2.05	0.59

NHIS-NSC CDM, National Health Insurance Service-National Sample Cohort-common data model; PS, propensity score.

^a^Results of a six-hospital CDM meta-analysis.

## Discussion

This study analyzed the incidence of CKD between PPI and H_2_RA users using large-scale healthcare databases, including the NHIS-NSC CDM and six-hospital CDM databases. The study employed large-scale PSM to balance the baseline characteristics of the two groups, and the results showed that PPI use was not associated with an increased risk of CKD compared to H_2_RA use.

PPIs are known to cause AKI by disrupting kidney function through several mechanisms. PPIs can reduce magnesium levels, which can interfere with the activity of enzymes and transporters in the kidneys, potentially leading to AKI. Additionally, PPIs can affect proton transport in the kidneys, which may also reduce kidney function and contribute to AKI. Furthermore, PPIs may increase the risk of ATIN, a condition that can cause AKI. In fact, several case reports have demonstrated a correlation between PPI use and ATIN^6–8^. However, it is believed that many patients with PPI-associated ATIN cases may not present with typical hypersensitivity reactions or undergo kidney biopsy, potentially leading to underdiagnosis of ATIN. This may lead to long-standing ATIN progressing to chronic tubulointerstitial nephritis, potentially contributing to CKD^18^. Although it is possible that long-standing ATIN may progress to chronic tubulointerstitial nephritis and contribute to CKD, this has not been directly proven by clinical studies. The mechanism by which PPIs may contribute to the development of CKD is not entirely clear. Therefore, conflicting results of clinical observational studies and unclear mechanisms make the association between long-term PPI use and CKD uncertain.

In the broader context of medical literature, findings regarding the association between PPI use and CKD risk have been mixed. Some studies have highlighted potential risks associated with PPIs, while others found no such associations^9,10,19,20^. Our Sensitivity Analysis, as presented in Table 3, indicates that patients with prolonged use of PPI (> 365 days) might have a lower propensity to develop CKD compared to those on H_2_RAs. However, this observation was not statistically significant. Notably, we have not identified studies that corroborate this specific observation. A possible explanation for this trend might be the influence of unmeasured confounding factors. For instance, patients on long-term PPI therapy might access medical care more frequently, leading to enhanced overall health monitoring and potentially early detection or prevention of CKD. Other factors, such as dietary habits, lifestyle choices, and unaccounted clinical variables, might also contribute to this observed trend.

Several epidemiologic studies suggested that PPIs could increase the risk of CKD. However, these studies had limitations. For instance, the PPI group had higher rates of comorbidities compared to the placebo group, and important information about CKD, such as baseline eGFR and concomitant medication, was not widely available for comparison between medication groups. Additionally, in studies comparing PPI use with H_2_RA use, it is unlikely that participants were well-matched for the severity of their gastrointestinal disorders since PPIs are often prescribed as a first-line therapy for more serious disorders such as Helicobacter pylori infection, gastroduodenal ulcers, and bleeding. Therefore, the positive signal toward CKD progression in PPI users may more accurately reflect a sicker group at baseline. Moreover, previous studies lacked the ability to determine the quantity and duration of PPI prescription use, which increases the risk of confounding during group assignments. This limitation is noteworthy because it can lead to the creation of alternative definitions of study outcomes.

This study represents a significant contribution to the literature examining the relationship between PPI use and CKD. This study addressed the limitations of previous research by utilizing large-scale healthcare databases and rigorous statistical methods to control for multiple confounding variables and the duration of PPI prescription. Notably, our subgroup analysis targeting patients with DM, consistently demonstrated that PPIs did not elevate the risk for CKD, even among diabetic individuals. Given the extensive size of our study and the significance of diabetes as a potential risk factor for CKD, this analysis offers crucial insights into the safety profile of PPIs in a high-risk demographic. However, concerns may arise regarding the representativeness of the study population due to the use of six-hospital CDM databases, possibly leading to selection bias and limiting the generalizability of the findings. While eGFR data availability in the six-hospital CDM databases is a strength, it is important to note that eGFR data was not available in the NHIS-CDM database, limiting the ability to diagnose CKD accurately in this dataset. Nevertheless, the study made the best use of both databases to overcome their respective weaknesses, providing a more comprehensive and robust analysis of the association between PPI use and CKD.

Despite the strengths of this study, there are some limitations that should be acknowledged.

Firstly, while the study controlled for multiple confounding variables, some unmeasured factors could affect the association between PPI use and CKD. For example, the study did not consider lifestyle factors such as smoking or alcohol consumption, which could potentially influence the risk of CKD. Secondly, the study design was based on comparing the incidence of CKD between PPI and H_2_RA users, which assumes that H_2_RA use does not contribute to CKD risk. If H_2_RA use were to have an impact on CKD risk, the study design might not accurately capture the true effect of PPI use on CKD risk. This could potentially lead to an underestimation of the association between PPI use and CKD. Thirdly, our study population did not include patients with established CKD; therefore, the effect of PPI use on this specific population remains unclear. Fourthly, our reliance on data up to 2013 and 2018 might not encapsulate the most recent shifts in clinical practices, drug formulations, or patient demographics. Fifthly, there were inherent differences between the groups before matching from the six hospitals, and a significant number of patients were excluded post-matching. This exclusion might limit the generalizability of our findings to the entire cohort of PPI and H2RA users. We have taken measures to balance the groups using propensity score matching, but the results should be interpreted with this limitation in mind. Lastly, it is important to note that in Korea, there is no evidence suggesting a bias in prescribing PPIs over H_2_RAs for early CKD, which might make the concept of protopathic bias less relevant to our study's context.

In conclusion, this study found no significant association between PPI use and an increased risk of CKD compared to H_2_RA use. Therefore, it is not recommended for clinicians to de-prescribe PPIs in patients who require continued therapy and are benefiting from it. However, there have been rare cases of acute tubulointerstitial nephritis (TIN), potentially leading to CKD, associated with PPI use. Providers need to individualize care to determine the benefits versus risks of ongoing medication use. Further research is needed to confirm these findings and investigate the potential mechanisms underlying the association between PPI use and CKD.

## Materials and methods

### Ethics statement

This study received ethical approval from the Institutional Review Board (IRB) at Kangdong Sacred Hospital, and the study was conducted in accordance with the principles of the Declaration of Helsinki. The requirement for written informed consent from study participants was waived by the IRB. The study was also conducted at six other hospitals that are affiliated with the Research Board Free Zone of the Korea CDM data network, which recognized the IRB approval of the research organizing center and did not require individual IRB approval for the study (Ref. no.2023-01-007).

### Data sources

This study utilized national population-based and hospital-based cohorts in the OMOP-CDM format for analysis. The primary analysis was conducted on the NHIS-NSC database, which contains medical treatment history, insurance eligibility, health examination findings, and healthcare provider information of over one million individuals. The NHIS-NSC database is a representative, stratified random sample of 2.2% of the Korean population in 2002, and the individuals were followed for 11 years. The OMOP-CDM version of the NHIS-NSC was validated in several multinational observational health data science and informatics studies through a common analytic R code^21–24^. The six hospital-based EHR databases were also transformed into the OMOP-CDM format and made accessible through the Federated E-health Big Data for Evidence Renovation Network in Korea (FEEDER-NET) (https://feedernet.com), supporting collaboration between OHDSI networks.

### Study design and cohort definition

This study utilized a retrospective observational design to compare the incidence of CKD in new users of PPIs and new users of H_2_RAs. To minimize the risk of immortal time bias, only patients with at least 365 days of a continuous observational period before entering either cohort were considered eligible for the study. The index date, marking the beginning of the study, was determined as the first date a patient used PPIs or H_2_RAs (Fig. 1).

Patients were included in the study if they met the following criteria: (1) New users of PPIs or H_2_RAs for over 180 days. New users were defined as those who did not use H_2_RAs within 365 days before starting PPI treatment and those who did not use PPIs within 365 days before starting H_2_RA treatment. (2) Aged 18 years or older. (3) Had an observation period of at least one year prior to cohort entry. (4) Had no previous history of glomerulonephritis (ICD-10 codes: N00-N08, M32.1, M32.14, M31.3, M31.31), kidney transplantation (ICD-10 codes: Z94.0, T86.1), or CKD (ICD-10 codes: N18.3-N18.5, N18.9).

The target cohort consisted of patients prescribed PPIs for a consecutive period of at least 180 days with no more than a 30-day gap between prescriptions. Medications in the target cohort included dexlansoprazole, esomeprazole, lansoprazole, omeprazole, pantoprazole, and rabeprazole. The comparative cohort comprised patients prescribed H_2_RAs for a consecutive period of at least 180 days with no more than a 30-day gap between prescriptions. The comparative cohort included ranitidine, nizatidine, famotidine, and cimetidine.

Patients were excluded from the study if they met any of the following criteria: 1) Had a diagnosis of CKD in NHIS-NSC CDM databases or an eGFR of less than 60 ml/min/1.73 m^2^ in six hospital-CDM databases. 2) Were not matched for the minimum time at risk of 1 day.

**Figure 4:**
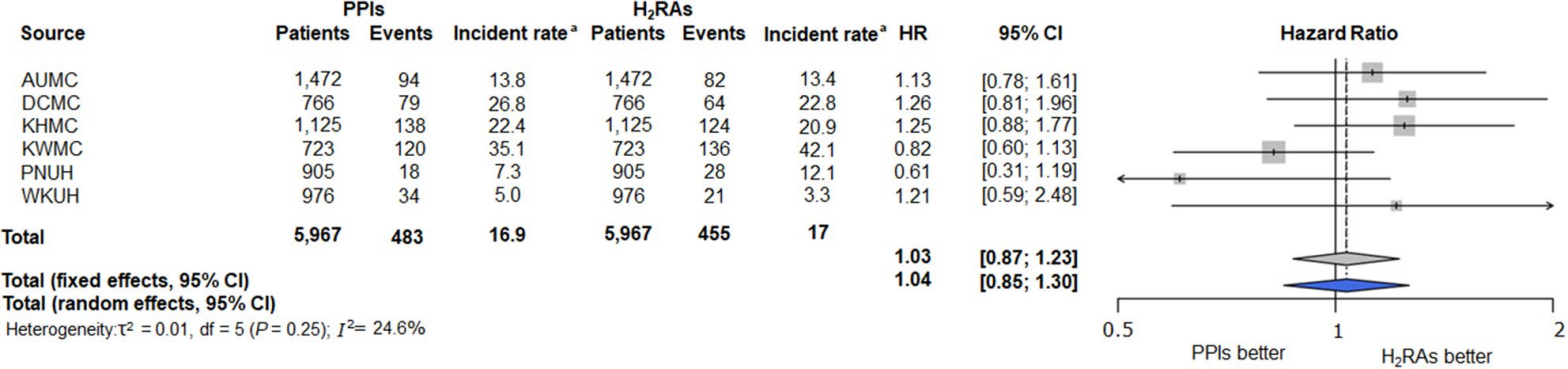
Incidence Rates of New-Onset Chronic Kidney Disease for PPI and H_2_RA Users with ≥ 180 Days of Use: Meta-Analysis Forest Plot. PPI, proton pump inhibitor; H_2_RA, histamine 2 receptor antagonist. ^a^Incidence rate per 1000 person-years.

In the six-hospital CDM databases, patients with eGFR below 60 ml/min/1.73 m^2^ were excluded from the analysis. It is important to note that eGFR data was not available in the NHIS-NSC CDM database but was available in the six hospital-CDM databases. The inclusion and exclusion criteria for the study are depicted in Fig. 4.

### Outcome

The outcome of this study was the occurrence of CKD, defined as having an eGFR of less than 60 ml/min/1.73 m^2^ or being diagnosed with ICD codes of N18.3-N18.5, N18.9. In the NHIS-NSC CDM database, CKD was defined using ICD codes, while in the six-hospital CDM databases, CKD was determined by having an eGFR less than 60 ml/min/1.73 m^2^ on at least three occasions during the observation period. The second eGFR measurement should be taken three months after the initial measurement of eGFR less than 60 ml/min/1.73 m^2^. The date of CKD diagnosis was based on the date of initial measurement.

### Statistical analysis

The study was conducted using the OHDSI CohortMethod R package (https://github.com/OHDSI/CohortMethod) and ATLAS version 2.7.6. To control for potential confounding, large-scale propensity score matching (PSM) was employed between the two groups. Covariates used in the matching process included age, sex, prior conditions, drugs observed during the long-term (within ~ 365 days) and short-term (within ~ 30 days) before study drug exposure, and the Romano Adaptation of the Charlson comorbidity index^25^.

To address overfitting, the investigators employed logistic regression models with an L1 penalty (also known as LASSO regularization) during the propensity score estimation. The L1 penalty serves to shrink some coefficients to zero, effectively performing feature selection and reducing the risk of overfitting. Moreover, the hyperparameter for the L1 penalty was chosen using tenfold cross-validation, a robust technique to ensure the model generalizes well to new data^26^. One-to-one greedy-search matching with a caliper of 0.2 times for the standard deviation of the propensity score distribution was used for matching patients.

The primary analysis was conducted with 1:1 propensity matching and a 180-day lag period. The 180-day lag period was chosen to minimize the potential for protopathic bias, wherein the initiation of the drug may be influenced by early symptoms of the outcome, in this case, CKD. This lag period allows for a more accurate assessment of the causal relationship between PPI or H_2_RA use and the development of CKD. During the analysis, patients who switched between PPIs and H2RAs were treated as censoring events to account for potential changes in treatment regimens. Cox regression was used to calculate the hazard ratio (HR) for CKD. Incidence rates were determined per 1000 person-years, and the cumulative incidence between the two groups was compared using the log-rank test. A two-sided P value of less than 0.05 was considered statistically significant. Empirical calibration of the P values was performed by fitting an empirical null distribution to the point estimates of the negative control outcomes, which were assumed not to be associated with the target or comparative cohorts^27,28^. The true relative risk between the target and comparative cohort was assumed to be 1. A total of 87 negative control outcomes were selected, listed in Supplementary Table S3.

After conducting the identical analytic process in six-hospital CDM databases, the results from each were combined using a meta-analysis. Statistical tests of heterogeneity were calculated using the tau-squared (τ^2^) and I^2^ statistics. When there was no significant difference between the results (P > 0.10, I2 < 50%), a fixed-effects model was used to combine the results. However, a random-effects model was used when there was a significant difference between the results. Both the fixed-effects and random-effects models were reported as a sensitivity analysis. All analyses were performed using R statistical software (version 3.6.1) provided by the R Foundation for Statistical Computing.

Additionally, sensitivity analyses were also conducted with different matching ratios (1:1 and 1:4) and lag periods (180 and 365 days) in propensity score matching. An analysis was also performed for patients with consecutive prescription periods ≥ 365 days to investigate potential dose–response effects. Furthermore, a subgroup analysis specifically targeted patients diagnosed with DM.

### Supplementary Information

Supplementary Information.

## Data Availability

CDM data are designed to support a distributed research network. Thus, access to the data is restricted on internal private networks. Therefore, data are not publicly available.
