# Supplementary material for: Proton pump inhibitors and chronic kidney disease risk: a comparative study with histamine-2 receptor antagonists
Source: Sci Rep. 2023 Dec 1;13:21169. doi: 10.1038/s41598-023-48430-9 (PMC10689439; doi:10.1038/s41598-023-48430-9)
Supplement: Supplementary file 1 — Supplementary Information. [file 41598_2023_48430_MOESM1_ESM.docx]

**Proton Pump Inhibitors and Chronic Kidney Disease Risk: A Comparative Study with Histamine-2 Receptor Antagonists**

Takhyeon Kweon, M.D., Yerim Kim, M.D., Kyung Joo Lee, B.S., Won-Woo Seo, M.D., Ph.D., Seung In Seo M.D., Ph.D., Woon Geon Shin M.D., Ph.D., Dong Ho Shin, M.D., Ph.D.*

Department of Internal Medicine, Kangdong Sacred Heart Hospital, Hallym University, College of Medicine, Seoul, Korea

Supplementary Table S1. The enrolled period and the number of individuals in NHIS-NSC CDM and six hospital-CDM databases used in the study.

| Database | Total number of participants | Study period |
| --- | --- | --- |
| NHIS-NSC CDM | 1,125,700 | 2002-2013 |
| Six-hospital CDM |  |  |
| AUMC | 3,109,677 | 1999-2018 |
| DCMC | 1,688,980 | 2005-2018 |
| KHMC | 2,010,456 | 2008-2018 |
| KWMC | 519,700 | 2003-2018 |
| PUNH | 1,753,001 | 2011-2018 |
| WKUH | 1,001,794 | 1998-2018 |

NHIS-NSC CDM, National Health Insurance Service National Sample Cohort Common Data

AUMC, Ajou University Medical Center; DCMC, Daegu Catholic Medical Center; KHMC, Kyung Hee University Medical Center; KWMC, Kangwon National University Hospital; PUNH, Pusan National University Hospital; WKUH, Wonkwang University Hospital

Supplementary Table S2. List of critical covariates considered in propensity score matching


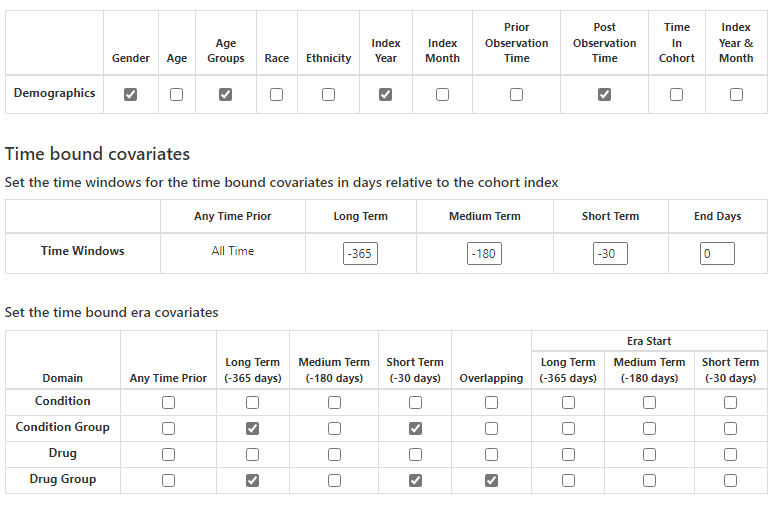


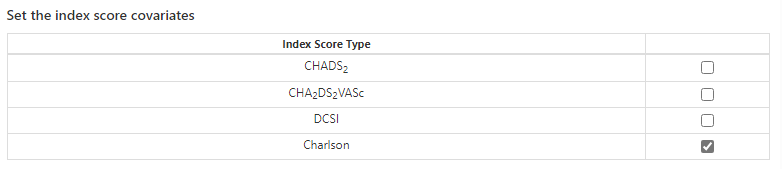


# Supplementary Table S3. List of negative control outcomes

| Concept ID | Concept code | Concept Name |
| --- | --- | --- |
| 4339088 | 87860000 | Testicular mass |
| 4322737 | 427898007 | Infection of tooth |
| 4307254 | 423125000 | Closed fracture |
| 4297984 | 76844004 | Local infection of wound |
| 4295888 | 76641005 | Prolapse of intestine |
| 4288544 | 396232000 | Inguinal hernia |
| 4285569 | 68633000 | Pupillary disorder |
| 4270490 | 62994001 | Tracheitis |
| 4242416 | 58588007 | Cutis laxa |
| 4215978 | 414941008 | Onychomycosis |
| 4207688 | 55184003 | Infectious enteritis |
| 4195698 | 67801009 | Tenosynovitis |
| 4172458 | 49883006 | Candidiasis of skin |
| 4171915 | 274718005 | Orchitis |
| 4171549 | 419153005 | Nodular goiter |
| 4163232 | 45198002 | Mastitis |
| 4153877 | 269406001 | Post-traumatic wound infection |
| 4153380 | 371160000 | Disorder of carotid artery |
| 4147672 | 30415006 | Disease due to Papilloma virus |
| 4146239 | 267802000 | Pruritus of genital organs |
| 4140510 | 3305006 | Disorder of lymphatic vessel |
| 4131595 | 12676007 | Fracture of radius |
| 4092565 | 24976005 | Uterine prolapse |
| 4047787 | 123971006 | Colles' fracture |
| 4047269 | 229844004 | Deformity of foot |
| 4029966 | 128609009 | Intracranial aneurysm |
| 4029582 | 237793004 | Hyperandrogenization syndrome |
| 4028970 | 13617004 | Tracheobronchitis |
| 4018050 | 10443009 | Localized infection |
| 444191 | 125593007 | Injury of face |
| 444130 | 125604000 | Injury of foot |
| 442274 | 52073004 | Oligomenorrhea |
| 441788 | 240532009 | Human papilloma virus infection |
| 440814 | 70070008 | Torticollis |
| 440695 | 302690004 | Encopresis |
| 440424 | 87486003 | Aphasia |
| 440389 | 91138005 | Mental retardation |
| 439840 | 1415005 | Lymphangitis |
| 439237 | 52684005 | Assault |
| 438872 | 267023007 | Excessive eating - polyphagia |
| 438407 | 78004001 | Bulimia nervosa |
| 438134 | 77692006 | Hypersomnia |
| 437409 | 127296001 | Intracranial injury |
| 436740 | 17382005 | Cervical incompetence |
| 436100 | 60380001 | Narcolepsy |
| 434630 | 3745000 | Sleep-wake schedule disorder |
| 434626 | 20010003 | Borderline personality disorder |
| 434319 | 44001008 | Premature ejaculation |
| 433440 | 78667006 | Dysthymia |
| 433163 | 238107002 | Deficiency of macronutrients |
| 381581 | 1482004 | Chalazion |
| 380731 | 3135009 | Otitis externa |
| 380395 | 314407005 | Retinal dystrophy |
| 378424 | 82649003 | Astigmatism |
| 378256 | 46670006 | Abnormal reflex |
| 378160 | 65668001 | Otorrhea |
| 376132 | 62909004 | Ectropion |
| 373478 | 41256004 | Presbyopia |
| 261880 | 46621007 | Atelectasis |
| 261326 | 75570004 | Viral pneumonia |
| 253796 | 36118008 | Pneumothorax |
| 199876 | 73998008 | Prolapse of female genital organs |
| 198075 | 240542006 | Condyloma acuminatum of the anogenital region |
| 195501 | 69878008 | Polycystic ovaries |
| 195212 | 47270006 | Hypercortisolism |
| 194997 | 9713002 | Prostatitis |
| 193874 | 8009008 | Nocturnal enuresis |
| 193326 | 87557004 | Urge incontinence of urine |
| 192606 | 60389000 | Paraplegia |
| 141825 | 267369002 | Simple goiter |
| 140641 | 57019003 | Verruca vulgaris |
| 140362 | 36976004 | Hypoparathyroidism |
| 139099 | 400097005 | Ingrowing nail |
| 137054 | 201066002 | Skin striae |
| 134765 | 238108007 | Cachexia |
| 134222 | 125597008 | Injury of forearm |
| 134118 | 400190005 | Atrophic condition of skin |
| 133228 | 80967001 | Dental caries |
| 133141 | 6020002 | Tinea pedis |
| 81336 | 57773001 | Rectal prolapse |
| 80509 | 203465002 | Bone cyst |
| 79072 | 266579006 | Inflammatory disorder of breast |
| 78804 | 27431007 | Fibrocystic disease of breast |
| 76737 | 55434001 | Hydrocele |
| 74855 | 33839006 | Genital herpes simplex |
| 73302 | 64217002 | Curvature of spine |

Supplementary Table S4. Baseline characteristics of PPI and H_2_RA groups with ≥180 days of use in Ajou University Medical Center.

|  | Before PS adjustment | |  | After PS adjustment | |  |
| --- | --- | --- | --- | --- | --- | --- |
| Characteristic (%) | PPIs | H_2_RAs | SMD | PPIs | H_2_RAs | SMD |
|  | (n=6,820) | (n=11,383) |  | (n=1,472) | (n=1,472) |  |
| Age group |  |  |  |  |  |  |
| 15-19 | 0.4 | 0.5 | -0.01 | 0.4 | 0.7 | -0.04 |
| 20-24 | 0.9 | 1.3 | -0.04 | 0.8 | 1.2 | -0.03 |
| 30-34 | 1.4 | 2.8 | -0.09 | 2.0 | 1.9 | 0 |
| 35-39 | 2.8 | 4.2 | -0.08 | 4.4 | 4.3 | 0 |
| 40-44 | 4.7 | 6.1 | -0.06 | 6.3 | 6.2 | 0.01 |
| 45-49 | 7.7 | 8.3 | -0.02 | 9.3 | 9.1 | 0.01 |
| 50-54 | 11.7 | 10.6 | 0.04 | 10.8 | 10.0 | 0.03 |
| 55-59 | 15.1 | 12.2 | 0.08 | 14.1 | 14.3 | -0.01 |
| 60-64 | 15.0 | 12.4 | 0.08 | 13.5 | 13.2 | 0.01 |
| 65-69 | 13.1 | 12.6 | 0.02 | 12.5 | 12.4 | 0 |
| 70-74 | 11.5 | 12.1 | -0.02 | 10.7 | 10.5 | 0.01 |
| 80-84 | 4.6 | 4.5 | 0.01 | 4.2 | 4.8 | -0.03 |
| 85-89 | 1.8 | 1.6 | 0.02 | 1.9 | 2.2 | -0.02 |
| Sex: female | 56.4 | 55.9 | 0.01 | 56.0 | 57.3 | -0.03 |
| Medical history |  |  |  |  |  |  |
| Diabetes mellitus | 10.5 | 10.3 | 0.01 | 10.6 | 10.3 | 0.01 |
| Gastroesophageal reflux disease | 9.4 | 3.1 | 0.26 | 8.5 | 7.8 | 0.02 |
| Hyperlipidemia | 5.3 | 5.6 | -0.01 | 5.6 | 5.8 | -0.01 |
| Hypertensive disorder | 22.2 | 24.1 | -0.04 | 21.9 | 23.5 | -0.04 |
| Visual system disorder | 6.4 | 6.6 | -0.01 | 6.5 | 6.2 | 0.01 |
| Cerebrovascular disease | 5.3 | 5.3 | 0 | 4.6 | 4.6 | 0 |
| Coronary arteriosclerosis | 13 | 9.7 | 0.10 | 11.9 | 12.3 | -0.01 |
| Heart disease | 21.8 | 20.5 | 0.03 | 22.7 | 23.8 | -0.03 |
| Ischemic heart disease | 14.0 | 12.3 | 0.05 | 15.0 | 15.8 | -0.02 |
| Malignant neoplastic disease | 5 | 9 | -0.16 | 6.5 | 6.4 | 0.01 |
| Medication |  |  |  |  |  |  |
| Agents acting on the renin-angiotensin system | 22.2 | 27.4 | -0.12 | 25.3 | 24.9 | 0.01 |
| Antibacterials for systemic use | 16.6 | 31.1 | -0.35 | 18.3 | 17.0 | 0.03 |
| Antiepileptics | 24 | 17.6 | 0.16 | 15.1 | 14.4 | 0.02 |
| Antiinflammatory and antirheumatic products | 76.6 | 67.5 | 0.21 | 67.4 | 68.8 | -0.03 |
| Antineoplastic agents | 25.7 | 19.1 | 0.16 | 20.5 | 20.0 | 0.01 |
| Antithrombotic agents | 46.0 | 47.7 | -0.03 | 45.2 | 47.9 | -0.05 |
| Drugs for acid related disorders | 24.9 | 58.2 | -0.72 | 35.3 | 32.5 | 0.06 |
| Drugs for obstructive airway diseases | 21.2 | 23.8 | -0.06 | 16.6 | 15.8 | 0.02 |
| Drugs used in diabetes | 10.5 | 12 | -0.04 | 9.9 | 10.8 | -0.03 |
| Immunosuppressants | 9.0 | 12.7 | -0.12 | 16.5 | 15.6 | 0.03 |
| Lipid modifying agents | 36.2 | 32.3 | 0.08 | 35.5 | 37.8 | -0.05 |
| Opioids | 36.9 | 35 | 0.04 | 29.1 | 29.0 | 0 |
| Psycholeptics | 22.5 | 36 | -0.30 | 24.3 | 22 | 0.05 |

PS, propensity score; PPI, proton pump inhibitor; H_2_RA, H_2_ receptor antagonist; SMD, standardized mean difference.

Supplementary Table S5. Baseline characteristics of PPI and H_2_RA groups with ≥180 days of use in Daegu Catholic Medical Center.

|  | Before PS adjustment | |  | After PS adjustment | |  |
| --- | --- | --- | --- | --- | --- | --- |
| Characteristic (%) | PPIs | H_2_RAs | SMD | PPIs | H_2_RAs | SMD |
|  | (n=3,151) | (n=8,485) |  | (n=766) | (n=766) |  |
| Age group |  |  |  |  |  |  |
| 15-19 | <0.2 | 0.1 | -0.01 | <0.7 | <0.7 | -0.03 |
| 20-24 | 0.3 | 0.6 | -0.04 | <0.7 | 1.2 | -0.11 |
| 25-29 | 0.4 | 0.6 | -0.03 | <0.7 | <0.7 | -0.02 |
| 30-34 | 0.5 | 0.8 | -0.04 | 0.8 | 1.2 | -0.04 |
| 35-39 | 1.2 | 1.6 | -0.03 | 1.6 | 1 | 0.05 |
| 40-44 | 2.1 | 3.2 | -0.07 | 2.3 | 2.5 | -0.01 |
| 45-49 | 4.8 | 5.6 | -0.04 | 5 | 4.2 | 0.04 |
| 50-54 | 8.7 | 8.8 | 0 | 9.5 | 7.8 | 0.06 |
| 55-59 | 12.4 | 12.9 | -0.01 | 11.9 | 14.4 | -0.07 |
| 60-64 | 16.7 | 14.3 | 0.07 | 16.2 | 14.6 | 0.04 |
| 65-69 | 14.8 | 15.2 | -0.01 | 14.8 | 15 | -0.01 |
| 70-74 | 15.3 | 15.4 | 0 | 15.5 | 16.3 | -0.02 |
| 75-79 | 13.0 | 12.4 | 0.02 | 12.4 | 10.7 | 0.05 |
| 80-84 | 7.2 | 6.1 | 0.04 | 6.5 | 6.8 | -0.01 |
| 85-89 | 2.1 | 2 | 0.01 | 2.1 | 2.7 | -0.04 |
| 90-94 | 0.3 | 0.4 | -0.02 | <0.7 | 0.7 | -0.05 |
| Sex: female | 52.8 | 58.9 | -0.12 | 52.9 | 54.8 | -0.04 |
| Medical history |  |  |  |  |  |  |
| Diabetes mellitus | 9.8 | 6.5 | 0.12 | 8.4 | 9.1 | -0.03 |
| Gastroesophageal reflux disease | 41.9 | 4.6 | 0.98 | 12.9 | 17.5 | -0.13 |
| Hyperlipidemia | 34.3 | 28.0 | 0.14 | 24.0 | 27.2 | -0.07 |
| Hypertensive disorder | 35.3 | 31.3 | 0.09 | 27.3 | 31.9 | -0.10 |
| Lesion of liver | 5.1 | 4.3 | 0.04 | 5.9 | 5.4 | 0.02 |
| Visual system disorder | 10.2 | 8.1 | 0.07 | 8.1 | 9 | -0.03 |
| Cerebrovascular disease | 16.2 | 16.1 | 0 | 8.0 | 9.8 | -0.06 |
| Heart disease | 26.6 | 23.2 | 0.08 | 37.9 | 38.8 | -0.02 |
| Heart failure | 5.1 | 6.5 | -0.06 | 6.0 | 7.6 | -0.06 |
| Ischemic heart disease | 17.8 | 13.6 | 0.11 | 27.4 | 27.0 | 0.01 |
| Malignant neoplastic disease | 13.0 | 9.5 | 0.11 | 13.3 | 12.4 | 0.03 |
| Agents acting on the renin-angiotensin system | 35.5 | 29.9 | 0.12 | 36.7 | 37.7 | -0.02 |
| Antibacterials for systemic use | 18.9 | 25.5 | -0.16 | 22.5 | 21.0 | 0.04 |
| Antidepressants | 12.5 | 16.5 | -0.11 | 12.3 | 12.8 | -0.02 |
| Antiepileptics | 10.5 | 16.6 | -0.18 | 11.2 | 11.4 | 0 |
| Antiinflammatory and antirheumatic products | 47.1 | 65.6 | -0.38 | 61.4 | 60.0 | 0.02 |
| Antineoplastic agents | 11.1 | 17.7 | -0.19 | 16.4 | 14.5 | 0.05 |
| Antithrombotic agents | 50.9 | 49.3 | 0.03 | 51.7 | 55.4 | -0.07 |
| Drugs for acid related disorders | 35.6 | 67.7 | -0.68 | 38 | 37.9 | 0 |
| Drugs for obstructive airway diseases | 15.6 | 33.2 | -0.42 | 19.7 | 19.2 | 0.01 |
| Drugs used in diabetes | 12.3 | 13.5 | -0.04 | 13.4 | 12.7 | 0.02 |
| Lipid modifying agents | 44.3 | 32.8 | 0.24 | 40.1 | 43.9 | -0.08 |
| Opioids | 21.2 | 26.7 | -0.13 | 26.5 | 24.4 | 0.05 |
| Psycholeptics | 38.6 | 35.7 | 0.06 | 31.6 | 33 | -0.03 |

PS, propensity score; PPI, proton pump inhibitor; H_2_RA, H_2_ receptor antagonist; SMD, standardized mean difference.

Supplementary Table S6. Baseline characteristics of PPI and H_2_RA groups with ≥180 days of use in Kyung Hee University Medical center.

|  | Before PS adjustment | |  | After PS adjustment | |  |
| --- | --- | --- | --- | --- | --- | --- |
| Characteristic (%) | PPIs | H_2_RAs | SMD | PPIs | H_2_RAs | SMD |
|  | (n=9,843) | (n=4,341) |  | (n=1,125) | (n=1,125) |  |
| Age group |  |  |  |  |  |  |
| 15-19 | 0.1 | 0.2 | -0.04 | <0.5 | <0.5 | 0.04 |
| 25-29 | 0.7 | 1.2 | -0.06 | 1.2 | 0.6 | 0.06 |
| 30-34 | 0.7 | 1.3 | -0.06 | 1.0 | 1.4 | -0.04 |
| 35-39 | 1.3 | 1.7 | -0.03 | 1.6 | 2.0 | -0.03 |
| 40-44 | 2.3 | 3.0 | -0.05 | 2.0 | 2.9 | -0.06 |
| 45-49 | 3.9 | 5.1 | -0.06 | 4.5 | 4.8 | -0.01 |
| 50-54 | 7.6 | 8.3 | -0.02 | 9.5 | 8.9 | 0.02 |
| 55-59 | 11.8 | 11.4 | 0.01 | 11.6 | 11.7 | -0.01 |
| 60-64 | 14.8 | 12.8 | 0.06 | 12.5 | 12.9 | -0.01 |
| 65-69 | 16.5 | 15.0 | 0.04 | 16.8 | 14.7 | 0.06 |
| 75-79 | 13.3 | 12.9 | 0.01 | 12.4 | 13.9 | -0.04 |
| 80-84 | 7.0 | 6.0 | 0.04 | 5.2 | 5.9 | -0.03 |
| 85-89 | 2.6 | 2.5 | 0.01 | 2.6 | 2.7 | -0.01 |
| 90-94 | 0.5 | 0.5 | 0.01 | 0.4 | 0.8 | -0.04 |
| Sex: female | 58.3 | 60.7 | -0.05 | 59.1 | 58.7 | 0.01 |
| Medical history |  |  |  |  |  |  |
| Depressive disorder | 6.4 | 9.0 | -0.10 | 6.9 | 9.1 | -0.08 |
| Diabetes mellitus | 12.5 | 11.8 | 0.02 | 12.9 | 12.5 | 0.01 |
| Gastroesophageal reflux disease | 33.7 | 7.1 | 0.70 | 11.6 | 15.9 | -0.12 |
| Hyperlipidemia | 38.8 | 30.6 | 0.17 | 28.9 | 31.6 | -0.06 |
| Hypertensive disorder | 38.3 | 41.2 | -0.06 | 39.5 | 38.3 | 0.02 |
| Osteoarthritis | 13.7 | 6.0 | 0.26 | 7.6 | 6.6 | 0.04 |
| Visual system disorder | 10.0 | 11.1 | -0.04 | 10.3 | 9.9 | 0.02 |
| Cerebrovascular disease | 6 | 9.9 | -0.14 | 6.8 | 5.4 | 0.06 |
| Heart disease | 29.8 | 21.8 | 0.18 | 28.0 | 27.3 | 0.02 |
| Ischemic heart disease | 21.5 | 14.1 | 0.20 | 20.4 | 19.5 | 0.02 |
| Malignant neoplastic disease | 6.5 | 8.1 | -0.06 | 7.6 | 7.8 | -0.01 |
| Agents acting on the renin-angiotensin system | 33.3 | 36.1 | -0.06 | 35.9 | 34.1 | 0.04 |
| Antibacterials for systemic use | 21.3 | 23.2 | -0.04 | 21.2 | 20.4 | 0.02 |
| Antidepressants | 16.4 | 19.0 | -0.07 | 17.4 | 16.6 | 0.02 |
| Antiinflammatory and antirheumatic products | 60.8 | 57.0 | 0.08 | 62.3 | 59.9 | 0.05 |
| Antineoplastic agents | 22.7 | 16 | 0.17 | 18.3 | 17.4 | 0.02 |
| Antithrombotic agents | 46.8 | 55.3 | -0.17 | 50.6 | 48.5 | 0.04 |
| Drugs for acid related disorders | 29.5 | 54 | -0.51 | 37.2 | 34.8 | 0.05 |
| Drugs for obstructive airway diseases | 22.6 | 23.6 | -0.02 | 24.4 | 22.8 | 0.04 |
| Drugs used in diabetes | 16.6 | 19 | -0.06 | 20.4 | 18.6 | 0.04 |
| Immunosuppressants | 5.4 | 6.5 | -0.05 | 7.8 | 6.1 | 0.07 |
| Lipid modifying agents | 45.6 | 43.1 | 0.05 | 44.4 | 43.1 | 0.02 |
| Opioids | 31.8 | 30.6 | 0.03 | 32.7 | 31 | 0.04 |
| Psycholeptics | 28.0 | 35.7 | -0.17 | 34.1 | 32.4 | 0.04 |

PS, propensity score; PPI, proton pump inhibitor; H_2_RA, H_2_ receptor antagonist; SMD, standardized mean difference.

Supplementary Table S7. Baseline characteristics of PPI and H_2_RA groups with ≥180 days of use in Kangwon National University Hospital.

|  | Before PS adjustment | |  | After PS adjustment | |  |
| --- | --- | --- | --- | --- | --- | --- |
| Characteristic (%) | PPIs | H_2_RAs | SMD | PPIs | H_2_RAs | SMD |
|  | (n=2,597) | (n=3,186) |  | (n=723) | (n=723) |  |
| Age group |  |  |  |  |  |  |
| 30-34 | 0.8 | 0.6 | 0.03 | <0.7 | <0.7 | -0.03 |
| 35-39 | 2 | 1.3 | 0.06 | 1.5 | 1.0 | 0.05 |
| 40-44 | 2.5 | 2.4 | 0.01 | 1.8 | 2.1 | -0.02 |
| 45-49 | 3.9 | 3.9 | 0 | 2.4 | 3.7 | -0.08 |
| 50-54 | 7.0 | 5.9 | 0.05 | 5.3 | 5.5 | -0.01 |
| 55-59 | 10.3 | 9.1 | 0.04 | 9.7 | 9.1 | 0.02 |
| 60-64 | 12.3 | 12.2 | 0 | 11.6 | 11.3 | 0.01 |
| 65-69 | 13.7 | 13.3 | 0.01 | 14.7 | 15.3 | -0.02 |
| 70-74 | 15.3 | 17.6 | -0.06 | 16.3 | 16.6 | -0.01 |
| 75-79 | 16 | 17.5 | -0.04 | 18.7 | 17.6 | 0.03 |
| 80-84 | 9.8 | 10.7 | -0.03 | 11.5 | 9.5 | 0.06 |
| 85-89 | 4.2 | 3.9 | 0.01 | 5.1 | 5.3 | -0.01 |
| 90-94 | 0.8 | 1 | -0.01 | 0.8 | 1.4 | -0.05 |
| Sex: female | 53 | 47.1 | 0.12 | 51.3 | 52.6 | -0.02 |
| Medical history |  |  |  |  |  |  |
| Hyperlipidemia | 22.6 | 13.7 | 0.23 | 17.4 | 20.2 | -0.07 |
| Hypertensive disorder | 20.3 | 17.1 | 0.08 | 20.7 | 21.2 | -0.01 |
| Visual system disorder | 10.9 | 12.5 | -0.05 | 9.5 | 8.9 | 0.02 |
| Atrial fibrillation | 6.8 | 4.2 | 0.11 | 7.7 | 7.3 | 0.02 |
| Cerebrovascular disease | 8.2 | 14.0 | -0.18 | 11.6 | 10.7 | 0.03 |
| Heart disease | 32.4 | 22.7 | 0.22 | 28.6 | 33.1 | -0.10 |
| Ischemic heart disease | 16.9 | 9.2 | 0.23 | 13.1 | 16.3 | -0.09 |
| Agents acting on the renin-angiotensin system | 30.3 | 29.9 | 0.01 | 35.3 | 33.9 | 0.03 |
| Antibacterials for systemic use | 21.1 | 34.0 | -0.29 | 25.0 | 23.2 | 0.04 |
| Antidepressants | 18.9 | 26.8 | -0.19 | 21.2 | 18.8 | 0.06 |
| Antiepileptics | 13 | 21.7 | -0.23 | 14.9 | 13.1 | 0.05 |
| Antiinflammatory and antirheumatic products | 69 | 74.1 | -0.11 | 67.6 | 64 | 0.08 |
| Antineoplastic agents | 20.7 | 10.6 | 0.28 | 10.8 | 11.8 | -0.03 |
| Antithrombotic agents | 51 | 59.3 | -0.17 | 59.2 | 54.9 | 0.09 |
| Drugs for acid related disorders | 31.5 | 47.4 | -0.33 | 32.1 | 30.4 | 0.04 |
| Drugs for obstructive airway diseases | 17.3 | 25.2 | -0.20 | 17.2 | 18.3 | -0.03 |
| Drugs used in diabetes | 16.5 | 16.8 | -0.01 | 18.1 | 15.4 | 0.07 |
| Lipid modifying agents | 38.8 | 35.8 | 0.06 | 41.2 | 38.2 | 0.06 |
| Opioids | 34.5 | 44.6 | -0.21 | 38.2 | 35.8 | 0.05 |
| Psycholeptics | 26.4 | 34.2 | -0.17 | 31.3 | 27.5 | 0.08 |

PS, propensity score; PPI, proton pump inhibitor; H_2_RA, H_2_ receptor antagonist; SMD, standardized mean difference.

Supplementary Table S8. Baseline characteristics of PPI and H_2_RA groups with ≥180 days of use in Pusan National University Hospital.

|  | Before PS adjustment | |  | After PS adjustment | |  |
| --- | --- | --- | --- | --- | --- | --- |
| Characteristic (%) | PPIs | H_2_RAs | SMD | PPIs | H_2_RAs | SMD |
|  | (n=2,994) | (n=3,022) |  | (n=905) | (n=905) |  |
| Age group |  |  |  |  |  |  |
| 20-24 | 0.7 | 1.4 | -0.07 | <0.6 | 1.3 | -0.11 |
| 25-29 | 1.2 | 0.9 | 0.02 | 1.2 | 0.9 | 0.03 |
| 30-34 | 1.3 | 1.2 | 0 | 1.2 | 1.7 | -0.04 |
| 35-39 | 1.8 | 2 | -0.02 | 2.2 | 2.1 | 0.01 |
| 40-44 | 3 | 2.9 | 0.01 | 3.0 | 2.3 | 0.04 |
| 45-49 | 5.3 | 4.9 | 0.02 | 5.4 | 4.4 | 0.05 |
| 50-54 | 7.6 | 9.1 | -0.06 | 7.8 | 8.6 | -0.03 |
| 55-59 | 12.8 | 12.5 | 0.01 | 12.4 | 12.9 | -0.02 |
| 60-64 | 17.3 | 15.6 | 0.05 | 17.7 | 16.7 | 0.03 |
| 65-69 | 16.8 | 15.5 | 0.04 | 15.5 | 16.6 | -0.03 |
| 70-74 | 14.8 | 15.8 | -0.03 | 15.2 | 14.8 | 0.01 |
| 75-79 | 11.1 | 11.5 | -0.01 | 11.5 | 11.7 | -0.01 |
| 80-84 | 4.8 | 4.7 | 0 | 4.6 | 4.5 | 0 |
| 85-89 | 1.3 | 1.5 | -0.02 | 1.5 | 1.0 | 0.05 |
| Sex: female | 51.5 | 48.6 | 0.06 | 49.8 | 48.7 | 0.02 |
| Medical history |  |  |  |  |  |  |
| Chronic liver disease | 4.2 | 6.1 | -0.08 | 7.2 | 6.4 | 0.03 |
| Diabetes mellitus | 10.6 | 13.2 | -0.08 | 11.4 | 9.9 | 0.05 |
| Hyperlipidemia | 10.1 | 13.4 | -0.10 | 9.4 | 8.5 | 0.03 |
| Hypertensive disorder | 16.3 | 17.3 | -0.02 | 14.4 | 14.6 | -0.01 |
| Lesion of liver | 5.4 | 6.6 | -0.05 | 7.4 | 6.5 | 0.04 |
| Visual system disorder | 7.5 | 7.0 | 0.02 | 7.1 | 5.6 | 0.06 |
| Heart disease | 18.5 | 19.8 | -0.03 | 20.9 | 23.5 | -0.06 |
| Ischemic heart disease | 11.3 | 11.3 | 0 | 12.2 | 13.6 | -0.04 |
| Malignant neoplastic disease | 20.3 | 18.3 | 0.05 | 17.1 | 15.8 | 0.04 |
| Agents acting on the renin-angiotensin system | 20.9 | 21.9 | -0.02 | 24.4 | 27.5 | -0.07 |
| Antidepressants | 18.9 | 15.6 | 0.09 | 13.7 | 14.1 | -0.01 |
| Antiepileptics | 9.0 | 14.2 | -0.16 | 11.4 | 9.7 | 0.05 |
| Antiinflammatory and antirheumatic products | 26.2 | 32.1 | -0.13 | 26.7 | 22.9 | 0.09 |
| Antithrombotic agents | 30.6 | 41.7 | -0.23 | 40.0 | 39.0 | 0.02 |
| Drugs for acid related disorders | 15.6 | 29.1 | -0.33 | 10.2 | 9.9 | 0.01 |
| Drugs for obstructive airway diseases | 12.1 | 9.6 | 0.08 | 9.4 | 9.9 | -0.02 |
| Drugs used in diabetes | 12.4 | 13.2 | -0.02 | 12.2 | 12.0 | 0 |
| Immunosuppressants | 10.5 | 9.2 | 0.04 | 7.7 | 7.8 | 0 |
| Lipid modifying agents | 28.4 | 32.9 | -0.10 | 33.4 | 35.0 | -0.04 |
| Opioids | 31.3 | 34.5 | -0.07 | 27.5 | 24.1 | 0.08 |
| Psycholeptics | 27.2 | 23.3 | 0.09 | 19.8 | 2.01 | -0.01 |

PS, propensity score; PPI, proton pump inhibitor; H_2_RA, H_2_ receptor antagonist; SMD, standardized mean difference.

Supplementary Table S9. Baseline characteristics of PPI and H_2_RA groups with ≥180 days of use in Wonkwang University Hospital

|  | Before PS adjustment | |  | After PS adjustment | |  |
| --- | --- | --- | --- | --- | --- | --- |
| Characteristic (%) | PPIs | H_2_RAs | SMD | PPIs | H_2_RAs | SMD |
|  | (n=6,636) | (n=5,975) |  | (n=976) | (n=976) |  |
| Age group |  |  |  |  |  |  |
| 15-19 | 0.2 | 0.2 | 0.01 | <0.5 | <0.5 | -0.06 |
| 20-24 | 0.6 | 0.4 | 0.03 | <0.5 | <0.5 | -0.04 |
| 25-29 | 0.9 | 0.6 | 0.03 | 0.6 | 1.1 | -0.06 |
| 30-34 | 1.4 | 0.6 | 0.07 | 2.0 | 1.1 | 0.07 |
| 35-39 | 2.4 | 1.5 | 0.06 | 1.9 | 2.2 | -0.01 |
| 40-44 | 4 | 3.4 | 0.03 | 2.3 | 3.2 | 0.01 |
| 45-49 | 6.4 | 5.7 | 0.03 | 7.1 | 5.9 | 0.05 |
| 50-54 | 10.3 | 10.0 | 0.01 | 9.6 | 10.9 | -0.04 |
| 55-59 | 13.4 | 12.2 | 0.04 | 12.4 | 11.8 | 0.02 |
| 60-64 | 14.4 | 14.7 | -0.01 | 13.8 | 15.0 | -0.03 |
| 65-69 | 14.0 | 15.3 | -0.04 | 15.9 | 12.8 | 0.09 |
| 70-74 | 11.5 | 14.6 | -0.09 | 13.5 | 14.2 | -0.02 |
| 75-79 | 11.1 | 12.7 | -0.05 | 10.8 | 11.5 | -0.02 |
| 80-84 | 6.5 | 6.1 | 0.02 | 6.8 | 6.7 | 0 |
| 85-89 | 2.3 | 1.8 | 0.04 | 1.4 | 2.4 | -0.07 |
| 90-94 | 0.6 | 0.3 | 0.05 | <0.5 | <0.5 | -0.02 |
| 95-99 | <0.1 | <0.1 | 0.00 | <0.5 | <0.5 | 0.03 |
| Sex: female | 55.4 | 53.2 | 0.04 | 53.1 | 54.5 | -0.03 |
| Medical history | |  |  |  |  |  |
| Diabetes mellitus | 10 | 11.9 | -0.06 | 10.0 | 10.2 | -0.01 |
| Hyperlipidemia | 12.4 | 23.2 | -0.29 | 18.1 | 16.4 | 0.05 |
| Hypertensive disorder | 31.2 | 45.8 | -0.30 | 35.9 | 33.8 | 0.04 |
| Lesion of liver | 6.9 | 3.8 | 0.13 | 7.2 | 5.9 | 0.05 |
| Visual system disorder | 7.5 | 9.3 | -0.06 | 8.6 | 8.5 | 0 |
| Cerebrovascular disease | 6.2 | 14.5 | -0.27 | 9.8 | 9.3 | 0.02 |
| Heart disease | 18.1 | 23.8 | -0.14 | 18.1 | 19.9 | -0.04 |
| Ischemic heart disease | 9.4 | 13.2 | -0.12 | 9.7 | 10.6 | -0.03 |
| Malignant neoplastic disease | 11.2 | 6.3 | 0.17 | 9 | 8.8 | 0.01 |
| Agents acting on the renin-angiotensin system | 22.3 | 37.8 | -0.34 | 28.8 | 25.7 | 0.07 |
| Antibacterials for systemic use | 28.6 | 32.1 | -0.08 | 23.1 | 21.5 | 0.04 |
| Antidepressants | 25.9 | 27.7 | -0.04 | 20.7 | 22.3 | -0.04 |
| Antiepileptics | 9.6 | 23.4 | -0.38 | 12.7 | 11.9 | 0.02 |
| Antiinflammatory and antirheumatic products | 50.5 | 58.8 | -0.17 | 46.0 | 44.9 | 0.02 |
| Antineoplastic agents | 23.4 | 9.0 | 0.40 | 9.6 | 11.4 | -0.06 |
| Antithrombotic agents | 28.8 | 55.9 | -0.57 | 39 | 36.9 | 0.04 |
| Drugs for acid related disorders | 55.2 | 76.8 | -0.47 | 61.2 | 58.5 | 0.05 |
| Drugs for obstructive airway diseases | 22.5 | 25.3 | -0.06 | 14.9 | 15.5 | -0.02 |
| Drugs used in diabetes | 11.8 | 17.1 | -0.15 | 14.0 | 14.1 | 0 |
| Lipid modifying agents | 24.2 | 35.0 | -0.24 | 29.3 | 30.3 | -0.02 |
| Opioids | 41.1 | 30.9 | 0.22 | 29.4 | 32 | -0.06 |
| Psycholeptics | 35.9 | 42.1 | -0.13 | 36.6 | 34.4 | 0.04 |

PS, propensity score; PPI, proton pump inhibitor; H_2_RA, H_2_ receptor antagonist; SMD, standardized mean difference.

| Database | Before PS adjustment | | |  | After PS adjustment | | |
| --- | --- | --- | --- | --- | --- | --- | --- |
|  | PPI | H2RA | SMD |  | PPI | H2RA | SMD |
| NHIS-NSC CDM | 4.3 | 3.6 | 0.24 |  | 4.3 | 4.2 | 0.02 |
| Six-hospital CDM |  |  |  |  |  |  |  |
| AUMC | 1.1 | 1.2 | -0.06 |  | 1.1 | 1.1 | 0.02 |
| DCMC | 1.8 | 1.5 | 0.17 |  | 1.6 | 1.7 | -0.09 |
| KHMC | 1.9 | 1.5 | 0.18 |  | 1.6 | 1.7 | -0.07 |
| KWMC | 1.5 | 1.5 | -0.03 |  | 1.5 | 1.4 | 0.04 |
| PUNH | 1.7 | 1.7 | 0.03 |  | 1.7 | 1.8 | -0.06 |
| WKUH | 1.6 | 1.6 | 0.01 |  | 1.8 | 1.7 | 0.04 |

Supplementary Table S10. Charlson comorbidity index score in NHIS-NSC CDM and six hospital-CDM databases used in the study

PS, propensity score; NHIS-NSC CDM, National Health Insurance Service National Sample Cohort Common Data Model; AUMC, Ajou University Medical Center; DCMC, Daegu Catholic Medical Center; KHMC, Kyung Hee University Medical Center; KWMC, Kangwon National University Hospital; PUNH, Pusan National University Hospital; WKUH, Wonkwang University Hospital; PPI, proton pump inhibitor; H_2_RA, H2 receptor antagonist; SMD, standardized mean difference.

Romano’s Adaptation of the Charlson Comorbidity Index was presented as the mean value.

**Supplementary Figure S1**. A plot of calibrated significance testing in the analysis in the NHIS-CDM database


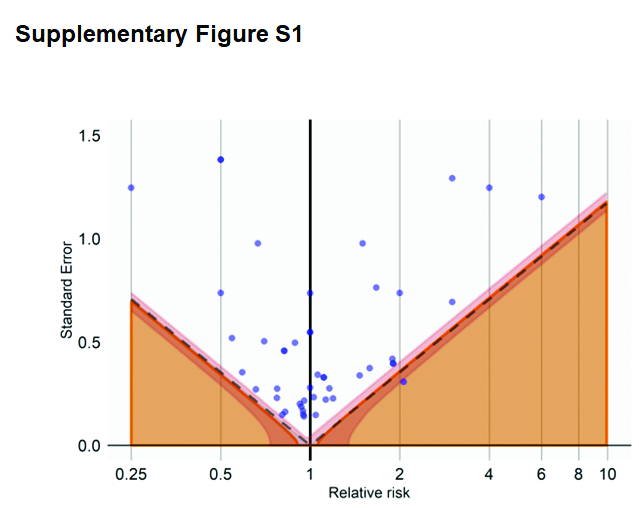


Blue dots indicate the estimates for negative control outcomes. The estimates in the orange areas have a P < 0.005 based on a calibrated P-value calculation.

NHIS-NSC CDM, National Health Insurance Service National Sample Cohort Common Data Model

**Supplementary Figure S2**. Distribution of propensity scores for study groups before and after matching in each dataset


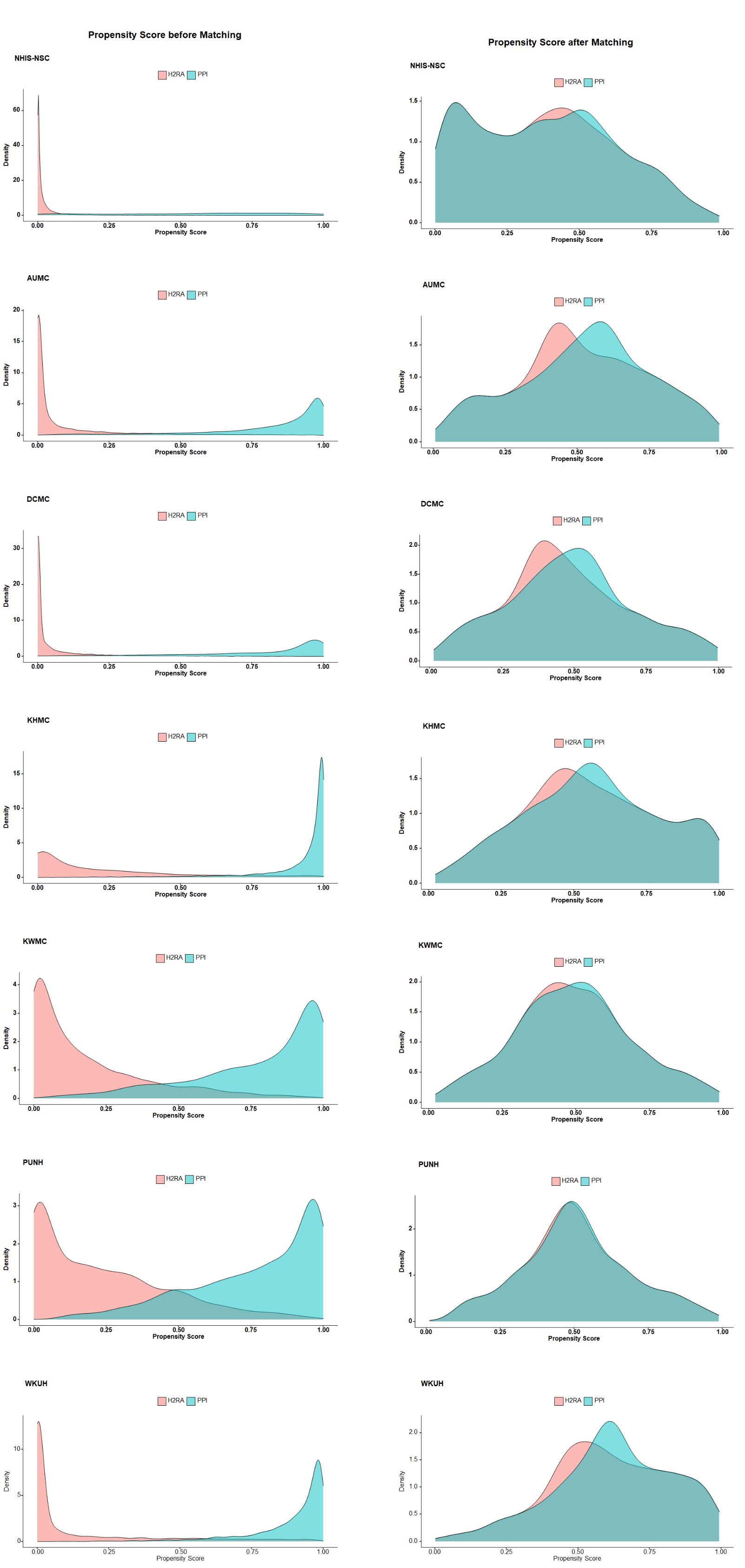


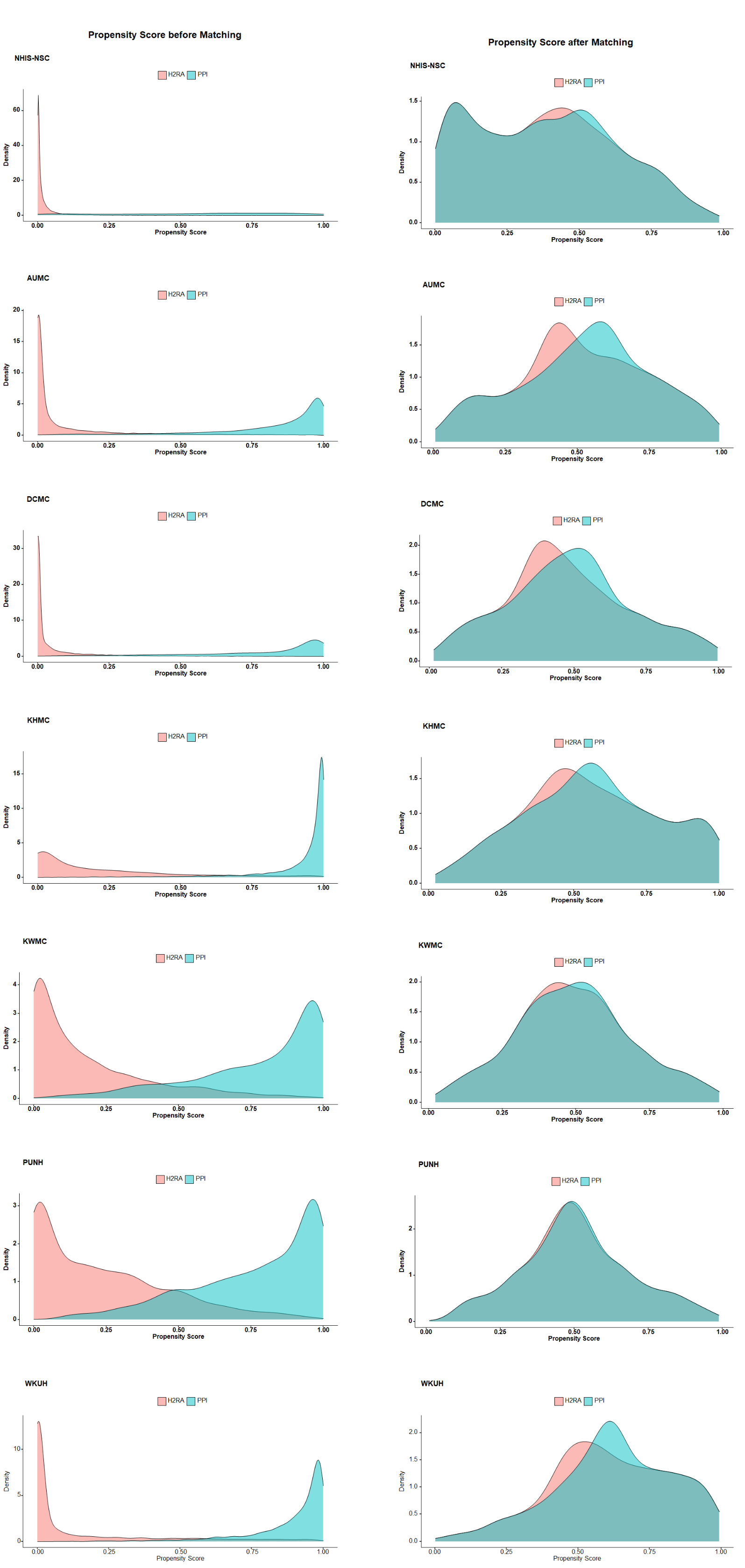


NHIS-NSC CDM, National Health Insurance Service-National Sample Cohort Common Data Model; AUMC, Ajou University Medical Center; DCMC, Daegu Catholic Medical Center; KHMC, Kyung Hee University Medical Center; KWMC, Kangwon National University Hospital; PUNH, Pusan National University Hospital; WKUH, Wonkwang University Hospital
